# Understanding Public Perceptions of the HPV Vaccination Based on Online Comments to Canadian News Articles

**DOI:** 10.1371/journal.pone.0129587

**Published:** 2015-06-08

**Authors:** Yael Feinberg, Jennifer A. Pereira, Susan Quach, Jeffrey C. Kwong, Natasha S. Crowcroft, Sarah E. Wilson, Maryse Guay, Yang Lei, Shelley L. Deeks, Public Health Agency of Canada/Canadian Institutes of Health Research Influenza Research Network (PCIRN) Program Delivery and Evaluation Group

**Affiliations:** 1 Public Health Ontario, Toronto, Canada; 2 Dalla Lana School of Public Health, University of Toronto, Toronto, Canada; 3 Institute for Clinical Evaluative Sciences, Toronto, Canada; 4 Department of Family and Community Medicine, University of Toronto, Toronto, Canada; 5 University Health Network, Toronto, Canada; 6 Laboratory Medicine and Pathobiology, University of Toronto, Toronto, Canada; 7 Département des sciences de la santé communautaire, Université de Sherbrooke, Longueuil, Canada; 8 Institut national de santé publique du Québec, Longueuil, Canada; 9 Centre de recherche de l’Hôpital Charles LeMoyne, Longueuil, Canada; Rudjer Boskovic Institute, CROATIA

## Abstract

**Background:**

Given the variation in human papillomavirus (HPV) vaccine coverage across Canada, and debate regarding delivery of HPV vaccines in Catholic schools, we studied online comments on Canadian news websites to understand public perceptions of HPV and HPV vaccine.

**Methods:**

We searched English- and French-language Canadian news websites for 2012 articles that contained the terms “HPV” or “human papillomavirus.” Articles about HPV vaccinations that contained at least one comment were included. Two researchers independently coded comments, analyzing them for emerging themes.

**Results:**

We identified 3073 comments from 1198 individuals in response to 71 news articles; 630 (52.6%) individuals expressed positive sentiments about HPV vaccination (2.5 comments/individual), 404 (33.7%) were negative (3.0 comments/individual), 34 (2.8%) were mixed (1.5 comments/individual) and 130 (10.8%) were neutral (1.6 comments/individual). Vaccine-supportive commenters believed the vaccine is safe and effective. Common themes in negative comments included concerns regarding HPV vaccine safety and efficacy, distrust of pharmaceutical companies and government, and belief that school-age children are too young for HPV vaccine. Many comments focused on whether the Catholic Church has the right to inform health policy for students, and discussion often evolved into debates regarding HPV and sexual behaviour. We noted that many individuals doubted the credibility of vaccine safety information.

**Conclusion:**

The majority of commenters do not appear to be against HPV vaccination, but public health messaging that focuses on both the vaccine’s safety profile, and its use as a means to prevent cancer rather than sexually transmitted HPV infection may facilitate its acceptance.

## Introduction

Human papillomavirus (HPV) is a common sexually transmitted infection that affects more than 70% of sexually active men and women at least once during their lifetime [1]. Within two to three years of sexual activity, 50–80% of adolescents will acquire HPV infection [2]. Recurrent HPV infection is associated with cervical, anogenital, and oropharyngeal cancers [1].

Two HPV vaccines, Gardasil and Cervarix, are approved for use in Canada for females and the former is also approved for males. Both vaccines protect against HPV 16 and 18, two strains that account for approximately 70% of cervical cancers. In Canada, all provinces and territories (P/Ts) have implemented publicly-funded school-based HPV vaccination programs for at least one school-grade cohort of females using Gardasil, with vaccine coverage of 50–85% [1]. However, some Catholic school boards have not permitted these clinics, generating heated debate [1,3–6]. In 2009, vaccine coverage was 18% in Calgary Catholic schools, which was largely due to the 2008 Calgary Catholic school board ban of HPV vaccination in its schools [4]. In defense of the ban, Bishop Frederick Henry of Calgary wrote in the National Post:

*If we don’t attempt to change sexual behaviour that is responsible for transmission of the HPV, but attempt to solve the problem by getting a series of shots, then we don’t have to exercise self-control, nor develop virtue, but can use medicine to palliate our vices [7].*

Following extensive debate, media coverage and public pressure from the advocacy group ‘HPV Calgary’ the ban in Calgary was rescinded in November 2012 [4]. The school board stated that they reconsidered the administration of the vaccine “in light of recent medical studies and developments, as well as consultation with our Bishopg [4]. Similar bans were also recently rescinded in Ontario (November 2013) and in Northwest Territories (May 2013) [8,9].

Given the controversy around HPV vaccine, and increasing vaccine hesitancy in general, awareness of societal opinion towards HPV vaccination is important in order for public health to develop targeted health promotion activities and messaging. Nearly 35% of Canadians frequent online newspaper sites regularly, many of which allow readers to post comments in reaction to articles [10]. Using our previous developed methodology [11], we evaluated reader responses to online newspaper articles to better understand Canadians’ perceptions of HPV and HPV vaccination.

## Methods

We conducted a descriptive qualitative study using online responses to news articles as our means of data collection. We identified relevant news articles by searching both national (n = 4) and P/T (n = 658) news sites listed on an online world newspaper website (http://www.onlinenewspapers.com).

We considered English and French news articles posted in 2012 that: 1) contained the terms *"HPV"* or *"human papillomavirus"* (“VPH” or “virus du papilloma human”) anywhere in the article or its title; 2) predominantly concerned HPV vaccine; and 3) generated at least one comment. Duplicate comments were excluded. Comments were moderated according to ‘Terms and Conditions’ listed by each newspaper, and removed if considered abusive or offensive.

## Analysis

We used the process of thematic analysis, which is a method for categorizing qualitative data. Thematic analysis uses codes to identify recurring topics to allow themes to emerge and was used due to its flexibility in analyzing large data sets [12]. Two researchers, J.A.P. and Y.F. developed a preliminary coding list consisting of topics frequently discussed in the comments. Using this coding list, J.A.P and Y.F. coded 10% of the comments independently. They continued to code an additional 20% of comments, conferring throughout to add to the coding dictionary. Inter-rater correlation on comment codes was very high (>98%). After consulting the study team to finalize this list, Y.F. coded the remaining 70% of comments. The codes were categorized into themes, which were reviewed to ensure accurate reflection of the data. Team members met regularly to review the data, and identify linkages between themes. The analysis was conducted in QSR NVivo 10 [13].

We followed a similar process to categorize comments by sentiment. J.A.P. and Y.F. independently reviewed 30% of comments, reaching consensus on each comment’s sentiment towards HPV vaccination: vaccination-supportive ("positive"), anti-vaccination ("negative"), conflicted feelings ("mixed"), or did not indicate an opinion ("neutral"). Y.F. then assessed sentiment for the remaining 70%. We summarized descriptive statistics, including the number of comments made across all comment boards per unique name, assuming each unique name represented a single individual. We recorded whether each comment included an HPV- or HPV vaccine-related statistic, a link to a website, or a personal story. We also noted other factors that may influence opinion of HPV and HPV vaccination, such as whether the individual self-identified as a healthcare worker (HCW), parent, or member of a particular religion.

Finally, we did a post hoc analysis to determine if there was a relationship between the sentiment of the article and the sentiment of the majority of the comments.

## Results

**Fig 1:**
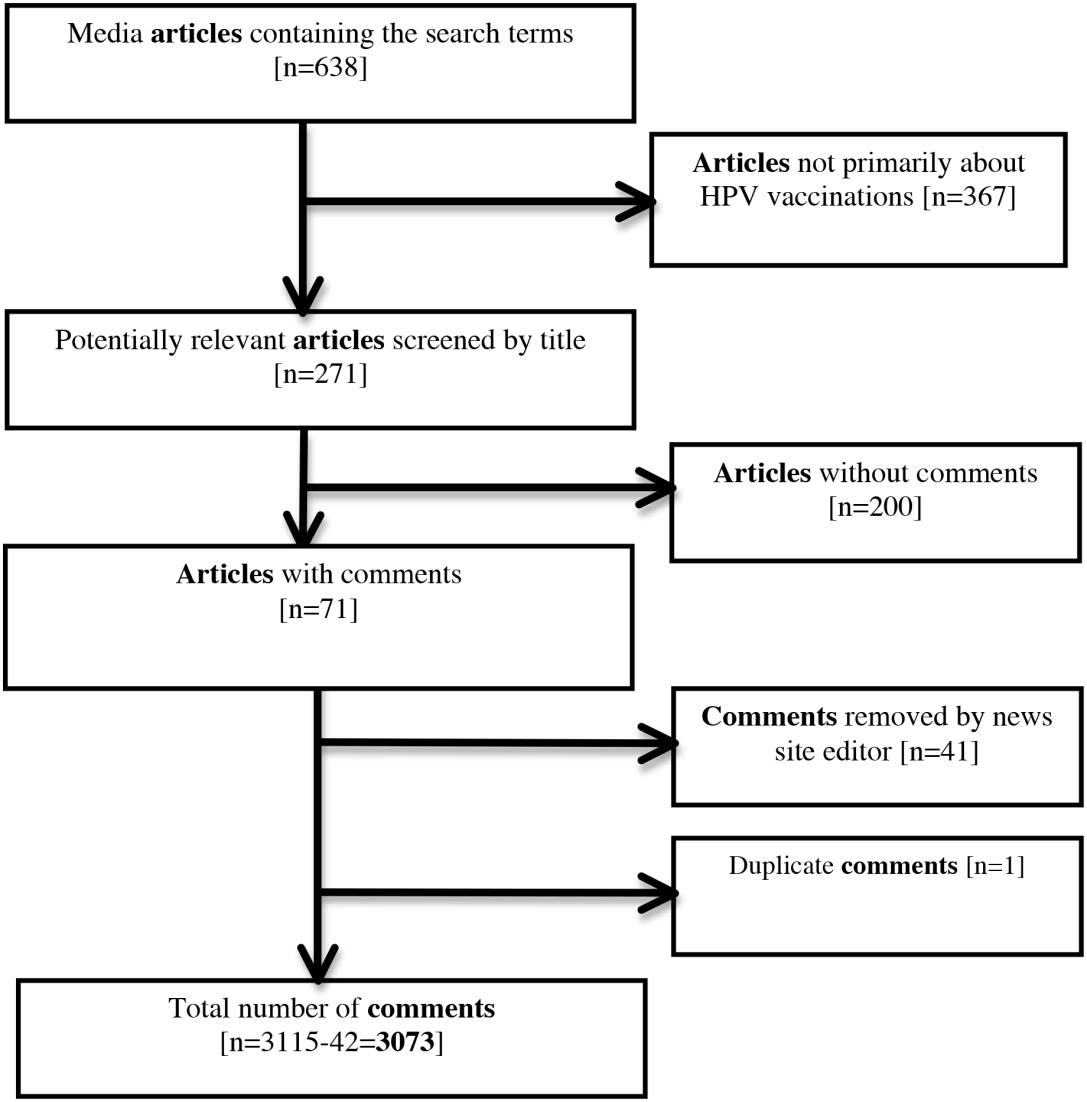
Flow diagram of inclusion and exclusion of articles and comments. This diagram illustrates the search and filter process used to identify the articles and comments.

**Fig 2:**
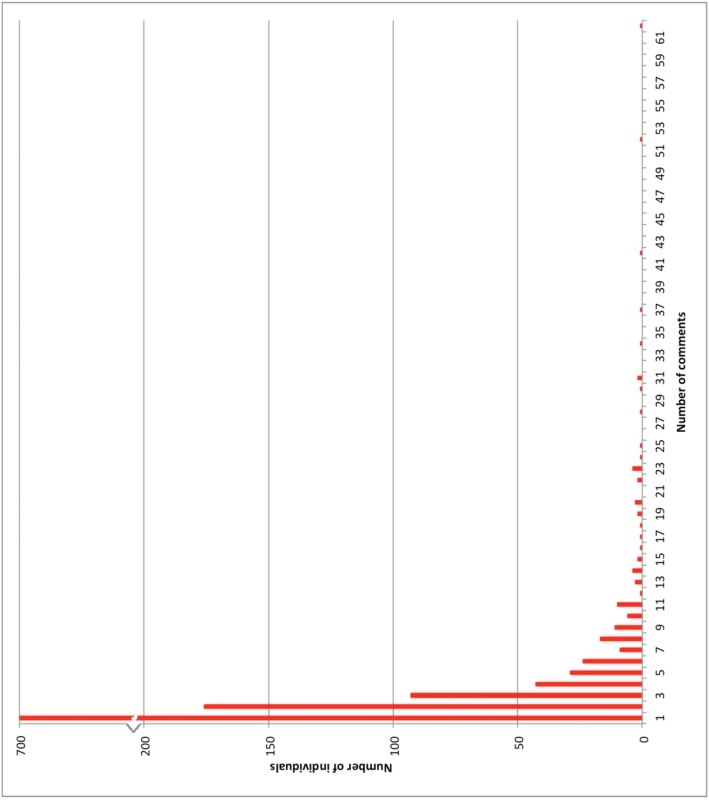
Histogram of number of comments by each individual. This histogram demonstrates the number of comments that individual commenters posted.

We identified 71 articles that met our inclusion criteria (Fig 1, Table 1). Of these, 52 were positive, 9 were negative, and 10 were neutral towards HPV vaccination (Table 2). The articles that were negative towards HPV vaccination were mostly from smaller local news sites. With respect to HPV vaccination at school, 13 articles were positive, 4 were negative, 26 were balanced towards HPV in school, and 28 did not discuss this topic specifically. The majority of articles (n = 59) were news features, while the remainder were opinion pieces. Thirty articles focused on the Calgary Catholic School Board controversy. In total, there were 3073 comments from 1198 individuals (median 1, mean 2.6, range: 1–62 comments/individual) (Table 3, Fig 2). Among commenters, 630 (52%) expressed positive perceptions of HPV vaccination (2.5 comments/individual), 404 (34%) were negative (3.0 comments/individual), 34 (3%) were mixed (1.5 comments/individual), and 130 (11%) were neutral (1.6 comments/individual) (Table 3). The articles that were negative were more likely to have a majority of negative comments, but these articles also attracted fewer total comments since the local papers reach a smaller audience (Table 2).

**Table 1:** Number of articles by province, including number of comments and individuals.

Province	Number of articles	Number of comments	Number of individuals
National	32	2158	863
Alberta	16	768	216
Ontario	15	79	67
Manitoba	5	49	35
British Columbia	2	16	14
Prince Edward Island	1	3	3
**Total**	**71**	**3073**	**1198**

**Table 2:** Sentiment of articles and sentiment of majority of comments.

		Sentiment of Articles		
		Positive	Negative	Neutral
**Sentiment of majority of comments**	**Positive**	32	2	6
	**Negative**	15	7	3
	**Equal**	5	0	1
	**Total**	52	9	10

**Table 3:** Descriptive statistics of individuals that commented on articles.

		Sentiment toward HPV vaccine			
	Total	Positive: n(%)	Negative: n (%)	Neutral: n(%)	Mixed: n(%)
Total number of individuals	1198	623 (52)	407 (34)	132 (11)	36 (3)
**Individual:**					
Provided link or statistic	152	52 (34)	96 (63)	3 (2)	1 (1)
Had personal story	61	36 (59)	24 (39)	0 (0)	1 (2)
Had HCW perspective	10	5 (50)	5 (50)	0 (0)	0 (0)
Parent perspective	104	42 (40)	57 (55)	0 (0)	5 (5)
Self-identified as Catholic	50	11 (22)	33 (66)	3 (6)	3 (6)

A total of 152 (13%) of all commenters provided links to information sources or statistics, the majority of which (63%) were anti-vaccination (Table 2). The most commonly cited sources by vaccine-supportive individuals were the U.S. Centers for Disease Control and Prevention (CDC), Wikipedia, and The Lancet. Anti-vaccine individuals linked to YouTube, Dr. Joseph Mercola’s natural health information site, and the CDC. Only 61 individuals (5.1%) provided a personal story about themselves or someone they knew involving HPV infection, cervical cancer, or a perceived adverse event temporarily associated with the vaccine, and 59% of these were vaccine-supportive. No common sentiment emerged from those who self-identified as a HCW or parent. Of the 50 individuals who self-identified as Catholic, the majority (66%) expressed negative sentiment.

**Fig 3:**
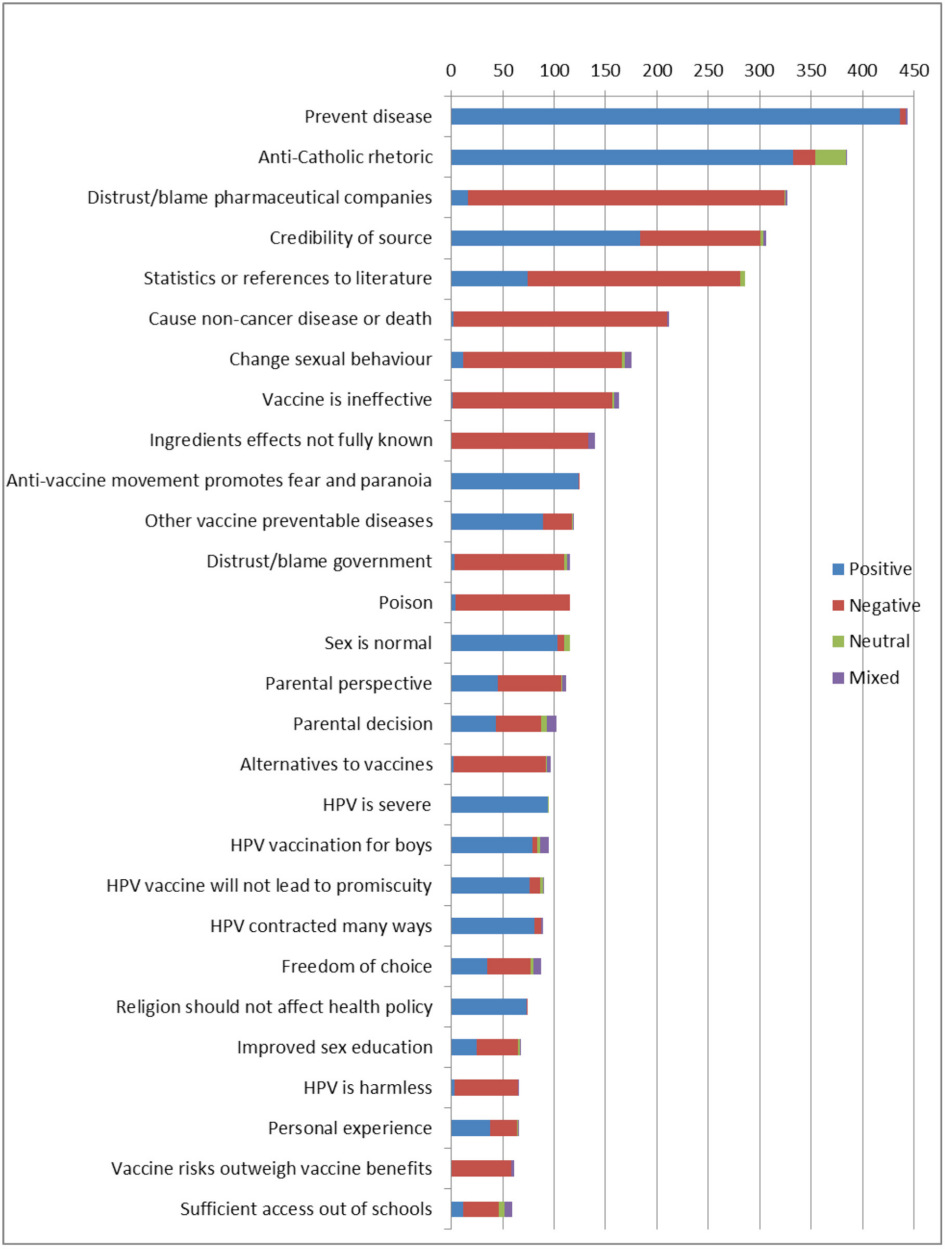
Common themes by sentiment. This diagram illustrates the most common themes discussed in the comments according to the sentiment of the comment.

Several themes were specific to vaccine-supportive or anti-vaccine comments (quantitative description in Fig 3, examples provided in Table 4). No recurring themes occurred across comments classified as mixed. Most HPV vaccine-neutral comments voiced criticisms of the Catholic Church but did not discuss vaccination issues.

**Table 4:** Comments associated with positive sentiment themes, negative sentiment themes and general themes.

Positive Sentiment Themes	*“Ever had HPV*, *dysplasia or squamous abnormalities*? *The testing you need if your GP finds any of those on your pap is awful and can feel very degrading*. *Believe it or not*, *the HPV shot can and will prevent lots of painful and invasive treatments*.*”* Velo Vixen, in response to http://www.theglobeandmail.com/life/health-and-fitness/health/why-some-parents-still-wont-give-daughters-the-hpv-vaccine/article4616841/
	*“The best way to address people concerned about vaccines is NOT to dismiss them as nutters but to address their fears with facts*. *Instead of just issuing blanket statements like "vaccines are perfectly harmless”[…] admit that there is a risk with every vaccination but the risk of an adverse reaction is*, *for example 0*.*01% but the risk of catching whooping cough and dieing (sp) without the vaccine is*, *for example 10%*.*”* pants7, in response to http://www.theglobeandmail.com/commentary/we-all-need-the-hpv-vaccine/article4375582/
	*“You teach your daughter to abstain before marriage and maybe she does*, *or maybe she does have sex*, *but always uses protection*. *She gets married and has unprotected sex with her husband*. *Oops—he had a previous partner and has now infected his wife with HPV and she develops cervical cancer*. *There is no amount of 'teaching' your child about safe sex that could prevent this from happening*. *Better to get vaccinated*.*”* Dawn302, in response to http://www.cbc.ca/news/canada/calgary/story/2012/06/25/calgary-hpv-vaccine-catholic-schools.html
Negative Sentiment Themes	*“I fully understand the disease and know that this HPV vaccine only addresses 4 of over 70 types*. *The fact remains that 90% of women can fight this off on their own within 2 years*. *My wife had HPV*, *how come she's not dead or had a hysterectomy*? *Now if you're asking me if I trust Merck enough to inject some of their crap in my daughter*, *the answer is no*.*”* justsayno in response to http://www.calgarysun.com/2012/11/28/calgary-catholic-school-district-approves-controversial-hpv-vaccinations
	*“By giving out Condemns (sp)*, *the pill or this Vaccine gives the idea that the church on one hand preach "save it till your married" while handing this crap out in schools*. *Makes it a complete contradiction*. *It suggests "do as we do*, *not as we say and creates a state of contradiction*. *Parents bare 100% responsibility for the vaccination of their children*. *As well individuals bare 100% responsibility for the choices they make*.*”* Jim_knuckles in response to http://www.calgarysun.com/2012/10/15/new-study-prompts-push-for-calgary-catholic-school-board-to-drop-ban-on-hpv-vaccine
	*“70% of all HPV infections resolve themselves without treatment within a year*. *Within two years*, *the number climbs to 90%*. *Of the remaining 10% of HPV infections*, *only half will develop into cervical cancer*, *which leaves little need for the vaccine*.*”* Opinion234, in response to http://www.cbc.ca/news/canada/story/2012/01/25/hpv-vaccine-males-gardasil.html
General Themes	*“OK folks*, *this is probably simpler than you might think… Who is most responsible and ultimately held accountable for a child's health and safety*? *That would be the parent or guardian*. *Definitely not a School Board*, *a Bishop*, *a HPV Crusader or a Policy Statement and most certainly not someone from within our checkered Alberta Health Care System*. *Regardless of how many letters are behind his/her name*.*”* ScottSOT, in response to http://www.calgarysun.com/2012/09/13/hpv-crusaders-set-to-sue-calgarys-catholic-school-board-over-anti-vaccination-stance
	*“TOTAL LIES AGAIN*!! *Do your research people before you believe garbage from mainstream media*. *I see from the posts people are realizing the truths about certain vaccines*, *you are all spot ON*. *There are numerous websites giving you the facts about the HPV which we must be aware of*, *the side effects alone are devastating*.*~ HEALTH SCIENCES INSTITUTE~ NATURALNEWS*.*COM~ DR*. *MERCOLA~ NATURAL SOCIETY~ MOTHER JONES~ INFOWARS*.*COM~ For the truth about natural cures for many (supposedly) uncureable (sp) diseases*.*”* pegasusXT, in response to http://www.theglobeandmail.com/commentary/we-all-need-the-hpv-vaccine/article4375582/
	*“And besides*, *a vaccine cannot replace PROPER SEXUAL EDUCATION*, *regarless (sp) of your position on vaccines*!*”* Siamese101, in response to http://www.cbc.ca/news/health/story/2012/04/13/hpv-vaccine-boys.html

### Positive sentiment themes

#### i) Prevention of HPV/cervical cancer

Many commenters discussed the value of prevention. Some cited the reduced disease burden of polio and smallpox as proof of vaccine effectiveness. In response to articles discussing HPV vaccination for boys, some remarked that the vaccine should be available to both sexes, given its ability to reduce cancer risk.

#### ii) Severity of HPV/cervical cancer

Many individuals commented on the severity of HPV infection and cervical cancer, acknowledging that treatment can be painful, unpleasant, and anxiety-provoking. Those with personal experiences with HPV infection or cancer expressed disbelief that anyone would refuse HPV vaccination.

#### iii) Vaccine benefits outweigh risks

Several vaccine-supportive individuals stated that the benefits of vaccination are well-proven in preventing cervical cancer, and outweigh any risk of adverse reactions from the vaccine.

#### iv) Cost-savings

In response to individuals claiming the HPV vaccine is too expensive for government to fund, many vaccine-supporters argued that by reducing disease burden, the vaccine saves healthcare dollars.

#### v) HPV contracted many ways/sex is normal

In response to commenters that advocated abstinence or fewer sexual partners as an alternative to the HPV vaccine, some commenters maintained that sexual activity is normal and that HPV can be acquired in ways beyond one’s control, for example through sexual assault and partners with undisclosed sexual history.

### Negative sentiment themes

#### i) Conspiracy

Many comments described a distrust of pharmaceutical companies, stating that they were either intentionally providing faulty vaccines to worsen health and create a dependence on medications, or manufacturing ineffective or unsafe vaccines for profit. Some individuals were also skeptical about what they perceived as the Canadian government’s fast-tracked approval of the HPV vaccine in 2006, allegedly due to pressure by pharmaceutical companies to endorse the vaccine. Others believed physicians and public health officials are linked with pharmaceutical companies and therefore pushing their agendas, or working with government to create fear in the public to promote vaccine uptake.

#### ii) Safety

Several commenters described vaccines as full of thimerosal, “toxins,” “poisons,” and undisclosed ingredients, the effects of which are unknown. Some individuals claimed that the HPV vaccine is carcinogenic, or causes chronic pain, fatigue, autism, blood clots, seizures, Guillain-Barré Syndrome, brain damage, infertility, and even death. Several commenters shared a personal story about an adverse reaction following a vaccine or medication. Individuals cited medications such as rofecoxib (Vioxx), thalidomide, and diethylstilbestrol (DES) that were eventually withdrawn due to adverse effects, further fuelling their distrust of pharmaceutical companies and of the government’s approval process for vaccines and medications.

#### iii) Efficacy

Many individuals stated that the vaccine is ineffective since it only covers four out of more than 100 HPV strains. Others claimed that not enough time has elapsed since vaccine introduction to determine its effectiveness in preventing cervical cancer. These people criticized studies claiming the HPV vaccine’s efficacy, arguing that an appropriate endpoint was cervical cancer instead of precancerous lesions. Others wrote that the vaccine is only effective for five years and that the current age of vaccination is too young.

#### iv) HPV is harmless/alternatives to vaccinations

Some commenters indicated HPV infection is harmless and cervical cancer is rare, concluding that risks of the vaccine outweigh any possible vaccine benefits. They stressed that in healthy people, HPV infections resolve without treatment, and that Pap smears and proper hygiene is sufficient to reduce cervical cancer incidence.

#### v) Change sexual behavior

Many of the negative comments claimed that HPV vaccination would be unnecessary if people changed their sexual behaviour. Suggestions included practicing abstinence, having fewer sexual partners, and using condoms. Many of these individuals also mentioned that changing sexual behaviour is preferable to the HPV vaccine as it will prevent more than just HPV infections.

### General themes

We noted several themes that were debated throughout the comment boards, but were not specific to those of a certain opinion of HPV vaccination.

#### i) Decision-making

Diverse comments centered on decision-making, some maintaining that parents alone should decide whether their children receive the HPV vaccine, while others stated vaccination decisions should be made by public health officials with the most knowledge. A few commenters indicated that adolescents themselves should make their own decisions.

#### ii) Credibility of source

Anti-vaccine comments claimed that the literature demonstrating vaccine safety and efficacy was biased due to pharmaceutical industry influence. Vaccine-supportive comments asserted that anti-vaccination sources promoted fear and paranoia, and were self-reported and non-reputable.

#### iii) HPV vaccine and promiscuity

Ten news articles referred to a study that did not find an association between HPV vaccination and promiscuity. In response, many expressed anger, indicating that this study wasted research money for what should have been obvious results. A number of anti-vaccine individuals agreed that the vaccine will not lead to promiscuity, but opposed the vaccine for reasons listed in the “Negative sentiment themes” section. Other anti-vaccine commenters stated their continued belief that the vaccine will promote promiscuity by giving the impression that sex is not dangerous.

#### iv) Improved sex education

Vaccine-supportive commenters expressed that parents should discuss the HPV vaccine, the limitations of Pap smears, and safe sex practices with their children. Those against the HPV vaccine stressed the importance of sexual education as an alternative to vaccination.

#### v) Appropriateness of school-based delivery of HPV vaccine

Many individuals, including some vaccine-supportive commenters, opposed school-based HPV vaccination programs, asserting that schools are not meant to provide medical care, and are not equipped to handle reactions that may arise post-vaccination. These commenters felt that sufficient access to the HPV vaccine exists outside of schools, and criticized HPV Calgary (a health advocate group promoting HPV vaccination) for opposing the Calgary Catholic School Board’s position. Some individuals considered HPV vaccinations in Catholic schools as problematic, citing their perception that the vaccine’s association with sexual behaviour contradicts the moral education students receive. Many of these commenters were only against HPV vaccination in Catholic schools, but supported the vaccine otherwise.

Conversely, many commenters were very supportive of school-based HPV vaccine delivery, claiming that schools are the most convenient and effective place to administer vaccinations for school-age children. In addition, some mentioned that the inconvenience of going to a clinic on three occasions for the complete vaccine schedule was a deterrent, particularly for at-risk populations, such as families of lower socioeconomic status. Many commenters stressed that religion should not affect health policy, and that since Catholic schools are publicly funded in Canada, Catholic schools should have to implement public health programs.

## Discussion

The topic of HPV vaccination in Canada involves myriad issues, and generated extensive media attention in 2012. Our results indicate that the majority of online commenters are supportive of HPV vaccination. However, anti-vaccine commenters are vocal in their opposition. This group is opposed to the vaccine for various reasons, including distrust of the pharmaceutical industry and government, safety and efficacy concerns regarding the vaccine, and the belief that vaccination is not necessary because HPV is harmless, or because effective alternatives to vaccination exist, such as changing sexual practices, improving hygiene, and bolstering sexual education. Other commenters were not opposed to the vaccine, but objected to vaccine delivery in Catholic schools.

Credibility of source was one of the most common themes across comment boards. Comments from various vaccine-supportive and anti-vaccine individuals expressed immense distrust of information sources. Nearly 13% of commenters referenced mistrust of vaccine-related data, including sources that are typically considered to be non-reliable, such as YouTube and Wikipedia, as well as more scientifically established sources such as the CDC. We also noted many inaccuracies across the comment boards, including the perceived severity of HPV, the link between HPV and cervical cancer, and the prevalence of cervical cancer. This suggests public health campaigns should strive to gain public trust and disseminate reliable information. It may be valuable for public health messaging to increase use of social media channels—which is not yet in regular practice [14]—to disseminate information and address misconceptions regarding the risks of HPV and cervical cancer [15].

Although most of the newspaper articles were positive towards HPV vaccination, nine articles were anti-vaccination. These negative articles were more likely to have a majority of comments opposed HPV vaccination. However, the anti-vaccination articles were mostly from smaller local news sites and attracted overall fewer comments. This indicates that news media have an important role in influencing public opinion regarding vaccination.

More than half of commenters who identified themselves as parents were against the vaccine, indicating that some parents may have concerns about the vaccine that have not yet been sufficiently addressed. One common theme expressed by commenters was freedom of choice regarding HPV vaccination, and that ultimately parents should decide whether their children are vaccinated. Future vaccination campaigns should emphasize that this is a voluntary program, and that parents should feel sufficiently informed before making a decision.

Ten newspaper articles referenced the study published in *Pediatrics* in October 2012 that debunked the purported association between HPV vaccination and promiscuity [16]. Interestingly, these articles elicited many angry comments labelling this study as a waste of research funds. While some commenters felt that the benefits of HPV vaccination could be easily achieved through abstinence and safe sexual practices, relatively few believed that HPV vaccination would promote promiscuity. HPV can be acquired though non-penetrative sexual touching, as well as through sexual behaviour outside an individual’s control (rape, unfaithful partners, etc.), and therefore, simply modifying sexual practices is not an effective substitute for vaccination [1]. Given the considerable debate associating HPV vaccination with sexuality and sexually transmitted infection, focusing messaging instead on the vaccine as an important tool for cancer prevention may facilitate its acceptance.

Traditionally, surveys have been used to collect information about health attitudes, but this method can be costly, untimely, and have limited generalizability. Our study is a notable contribution to data collected from social media to analyze public perceptions of vaccinations [1,17–19]. To our knowledge, two Canadian studies and one British study specifically analyzed online comments newspaper articles [11,17,20]. The advantages of analyzing perception data derived from social media is that they are current, readily available, and can be drawn from a large geographical area [20]. Additionally, the anonymous format of online messaging may allow commenters to be more forthcoming about true opinions rather than expressing what is socially acceptable. However, as with other types of perception tools such as key informant interviews and focus groups, those who participate are likely to have strong opinions on the topic discussed and thus may not be representative of the general population. Anonymity may also allow respondents to express themselves differently than they might in face-to-face interactions. Our study findings corroborate those found in previous analyses of both conventional and social media data, specifically MySpace blogs, which found diverse perspectives regarding HPV infection, vaccine safety and efficacy, and alternatives to vaccination [19,21].

There are a number of limitations of our study. We used comments in response to news articles as our sole source of public perception data. Future studies should consider alternate social media forms such as Facebook, Twitter, and blogs, to confirm findings. Our search strategy may have missed some relevant news articles from smaller, local news outlets. The demographics of commenters are unknown, and given the electronic medium allowing responses from anywhere, we are uncertain about the representativeness of these results to the wider Canadian public. Those who chose to comment online may have differing opinions than the general population; studies using other methods are needed to verify findings. Finally, while we assumed that every unique commenter name represented a single individual, some individuals may have used multiple names while commenting on the articles. Therefore, we might have overestimated the number of commenters, which also impacts the generalizability of our results.

## Conclusions

Our study demonstrated that the majority of commenters in our analysis were supportive of the HPV vaccine, although a substantial group remains opposed. Misinformation regarding the vaccine’s risks and benefits, as well as the severity of both HPV disease and cervical cancer, was common on the online comment boards. Public health messaging that focuses on both the HPV vaccine’s safety profile, and its use as a means to prevent cancer rather than sexually transmitted HPV infection may facilitate its acceptance.

## Supporting Information

S1 Table Number of comments and number of individuals commenting on each article. (DOCX)

S1 Dataset Spreadsheet of comments posted by username to all articles. (XLSX)
