# Supplementary material for: Understanding Public Perceptions of the HPV Vaccination Based on Online Comments to Canadian News Articles
Source: PLoS One. 2015 Jun 8;10(6):e0129587. doi: 10.1371/journal.pone.0129587 (PMC4460033; doi:10.1371/journal.pone.0129587)
Supplement: S1 Table — (DOCX) [file pone.0129587.s001.docx]

**S1 Table. Number of comments and number of individuals commenting on each article.**

| News source | Province | Date | Title | Archived link | Number of comments | Number of individuals |
| --- | --- | --- | --- | --- | --- | --- |
| The Guardian - Charlottetown Newspaper | PEI | 25-Jan-12 | HPV vaccine recommended for boys | <http://www.webcitation.org/6J2V9fdFE> | 3 | 3 |

| Georgia Straight | BC | 31-Oct-12 | UBC researchers advocate HPV vaccine scrutiny | <http://www.webcitation.org/6J2VGXCuV> | 6 | 14 |
| --- | --- | --- | --- | --- | --- | --- |
|  |  | 28-Nov-12 | Skeptic raises an alarm about HPV vaccinations | <http://www.webcitation.org/6J2W4YCJ4> | 10 |  |
| Winnipeg Free Press | MB | 25-Oct-12 | Calgary Catholic schools review policy against student HPV vaccinations | http://www.webcitation.org/6J2Y3C4Ea | 2 | 28 |
|  |  | 7-Feb-12 | Female physicians urge provinces, territories to pay for HPV vaccine for boys | http://www.webcitation.org/6J2YAbCJU | 34 |  |
|  |  | 24-Jan-12 | Gentlemen, roll up your sleeves: HPV vaccine recommended for boys | <http://www.webcitation.org/6J2ZHHL8q> | 5 |  |
| Winnipeg Sun | MB | 13-Sept-12 | Proponents may sue Calgary Catholic school board over HPV vaccination | <http://www.webcitation.org/6J2ZMgpDv> | 3 | 7 |
|  |  | 15-Oct-12 | HPV vaccine doesn't make young girls more promiscuous: Study | <http://www.webcitation.org/6J2ZRYNkD> | 5 |  |
| Calgary Herald | AB | 26-Oct-12 | Bishop supports HPV consultation, but still backs vaccine ban in schools | <http://www.webcitation.org/6J2ZccbUM> | 20 | 14 |
|  |  | 25-Oct-12 | Catholic schools to consult parents on HPV vaccine | http://www.webcitation.org/6J2ej554E | 7 |  |
|  |  | 13-Sept-12 | Catholic school may face legal challenge over refusal to offer HPV vaccine | http://www.webcitation.org/6J2erJ2jP | 3 |  |
| Calgary Sun | AB | 28-Nov-12 | Calgary Catholic School District approves controversial HPV vaccinations | http://www.webcitation.org/6J2eyjaxJ | 145 | 161 |
|  |  | 25-Oct-12 | Calgary Bishop Fred Henry backs policy review for HPV vaccinations in separate schools | <http://www.webcitation.org/6J2f883pM> | 93 |  |
|  |  | 13-Sept-12 | HPV crusaders set to sue Calgary's Catholic school board over anti-vaccination stance | http://www.webcitation.org/6J2fFsovT | 123 |  |
|  |  | 29-Nov-12 | Bishop disappointed in outcome of Catholic school board HPV vaccination vote | http://www.webcitation.org/6J2fLwjIO | 197 |  |
|  |  | 15-Oct-12 | Study sparks renewed push for Calgary Catholic trustees to allow HPV vaccine in schools | <http://www.webcitation.org/6J2fRON0t> | 13 |  |
|  |  | 15-Oct-12 | New study prompts push for Calgary Catholic school board to drop ban on HPV vaccine | <http://www.webcitation.org/6J2fYDU4K> | 81 |  |
|  |  | 24-Oct-12 | Calgary Catholic School District board pass motion to consult with parents about HPV shot | http://www.webcitation.org/6J2fddadC | 28 |  |
|  |  | 4-July-12 | Letters to the editor: July 4 | http://www.webcitation.org/6J2flawEI | 37 |  |
|  |  | 1-July-12 | Platt: Serious ethical flaw exposed | http://www.webcitation.org/6J2frfynW | 3 |  |
| Edmonton Sun | AB | 15-Oct-12 | HPV vaccine doesn't make young girls more promiscuous: Study | http://www.webcitation.org/6J2fzrQTb | 12 |  |
|  |  | 13-Sept-12 | Proponents may sue Calgary Catholic school board over HPV vaccination | http://www.webcitation.org/6J2jzUYrh | 2 |  |

| CTV Calgary News | AB | 29-Nov-12 | Calgary Catholic board will offer HPV vaccine | http://www.webcitation.org/6J2p2fB3y | 3 | 4 |
| --- | --- | --- | --- | --- | --- | --- |
|  |  | 28-Nov-12 | Catholic school board to vote on HPV | http://www.webcitation.org/6J2p56X4l | 1 |  |

| Toronto Sun | ON | 15-Oct-12 | HPV vaccine doesn't make young girls more promiscuous: Study | <http://www.webcitation.org/6J2k73K9V> | 10 | 21 |
| --- | --- | --- | --- | --- | --- | --- |
|  |  | 31-Jan-12 | Family sues after teen dies following HPV vaccination | <http://www.webcitation.org/6J2kES91O> | 5 |  |

|  |  | 13-Sept-12 | Proponents may sue Calgary Catholic school board over HPV vaccination | <http://www.webcitation.org/6J2kIKuUA> | 9 |  |
| --- | --- | --- | --- | --- | --- | --- |
| Ottawa Sun | ON | 15-Oct-12 | HPV vaccine doesn't make young girls more promiscuous: Study | <http://www.webcitation.org/6J2kMMmOj> | 7 | 6 |
| Durham Region | ON | 30-Jun-12 | Whitby mom shares daughter's story | <http://www.webcitation.org/6J2qCJXH4> | 4 | 15 |

|  |  | 16-Nov-12 | Know all the angles when it comes to vaccinations | <http://www.webcitation.org/6J2r2aDUB> | 1 |  |
| --- | --- | --- | --- | --- | --- | --- |
|  |  | 26-Jun-12 | Vaccine a sore spot for Whitby teen | <http://www.webcitation.org/6J2r7KLpo> | 17 |  |

| Belleville Intelligencer | ON | 28-Aug-12 | Vaccination expands | <http://www.webcitation.org/6J2rFhMpX> | 4 | 4 |
| --- | --- | --- | --- | --- | --- | --- |
| Brantford Expositor | ON | 30-Aug-12 | HPV vaccination program extended | http://www.webcitation.org/6J2rJIGlP | 2 | 2 |
| Hamilton Spectator | ON | 8-Nov-12 | Halton offers more HPV clinics | http://www.webcitation.org/6J2rWuqj9 | 3 | 3 |
| Northumberland News | ON | 22-Oct-12 | Say no to HPV vaccine | <http://www.webcitation.org/6J2s5FdoH> | 2 | 2 |
| North Bay Nugget | ON | 13-Sept-12 | Proponents may sue Calgary Catholic school board over HPV vaccination | http://www.webcitation.org/6J2s9992w | 1 | 1 |
| The Peterborough Examiner | ON | 13-Sept-12 | Proponents may sue Calgary Catholic school board over HPV vaccination | <http://www.webcitation.org/6J2sDSm0o> | 1 | 1 |
| The Record | ON | 29-Jun-12 | HPV vaccine ban in schools a dangerous precedent | http://www.webcitation.org/6J2sG32kt | 6 | 6 |
| SooToday.com | ON | 5-Nov-12 | HPV (Human Papillomavirus) immunization program expands | <http://www.webcitation.org/6J2sJdS1G> | 7 | 7 |

| CBC | National | 15-Apr-12 | Should boys have access to free HPV shots? | <http://www.webcitation.org/6J2lgvLkA> | 48 | 418 |
| --- | --- | --- | --- | --- | --- | --- |
|  |  | 28-Nov-12 | Calgary Catholic school trustees approve HPV vaccine | <http://www.webcitation.org/6J2lkvYB7> | 25 |  |
|  |  | 7-Nov-12 | Catholic parents weigh in on HPV vaccine for Calgary students | <http://www.webcitation.org/6J2mmNH7B> | 14 |  |
|  |  | 15-Oct-12 | HPV shots don't make girls promiscuous, study says | http://www.webcitation.org/6J2mpcl17 | 67 |  |
|  |  | 15-Apr-12 | Free HPV vaccine urged for boys | <http://www.webcitation.org/6J2msW6Re> | 317 |  |
|  |  | 25-Jan-12 | HPV vaccine for boys recommended in Canada | <http://www.webcitation.org/6J2n5C1zY> | 368 |  |
|  |  | 25-Jun-12 | Calgary group pushing for HPV vaccine in Catholic schools | <http://www.webcitation.org/6J2nBeyR3> | 32 |  |
|  |  | 21-Mar-12 | Sexually transmitted infections can hide, experts warn | http://www.webcitation.org/6J2nGat5P | 7 |  |
|  |  | 28-Jan-12 | P.E.I. considers free HPV vaccine for boys | <http://www.webcitation.org/6J2nJvPV7> | 4 |  |
| Globe and Mail | National | 28-Jun-12 | We all need the HPV vaccine | http://archive.is/hXUW5 | 305 | 218 |
|  |  | 28-Dec-12 | I’m so confused by HPV. Should my daughter get the vaccine? | http://archive.is/ua703 | 4 |  |
|  |  | 29-Nov-12 | Calgary Catholic schools finally lift ban on HPV vaccine | http://archive.is/SUhdl | 22 |  |
|  |  | 20-Nov-12 | Calgary Catholic school board should reverse ban on HPV vaccine | http://archive.is/wP8H3 | 35 |  |
|  |  | 5-Nov-12 | Catholic school boards should make HPV vaccine available to girls | http://archive.is/mx0oK | 28 |  |
|  |  | 25-Oct-12 | Calgary’s Catholic schools review ban on HPV vaccine | http://archive.is/GvMe5 | 7 |  |
|  |  | 15-Oct-12 | HPV shots don’t lead to increase in teen promiscuity, study finds | http://archive.is/FrlLi | 7 |  |
|  |  | 8-Feb-12 | Female physicians urge provinces to pay for HPV vaccine for boys | http://archive.is/VXGFF | 18 |  |
|  |  | 25-Jan-12 | Federal panel recommends HPV vaccine for boys | http://archive.is/1jtwL | 8 |  |
|  |  | 4-Jan-12 | HPV vaccine could deter safe sex, study finds | http://archive.is/of1L1 | 7 |  |
|  |  | 16-Oct-12 | Why some parents still won't give daughters the HPV vaccine | http://archive.is/QpS4K | 195 |  |
|  |  | 17-Sept-12 | Toronto Public Health expands free HPV vaccine program for teen girls | http://archive.is/ig1i7 | 15 |  |
| CTV News | National | 26-June-12 | Calgary Catholic school board urged to revisit HPV vaccine | <http://www.webcitation.org/6J2oXFpgs> | 16 | 28 |
|  |  | 15-Oct-12 | Vaccine for HPV doesn't make girls promiscuous: Study | http://www.webcitation.org/6J2oeN9se | 13 |  |
| National Post | National | 15-Oct-12 | Vaccine against cancer-causing HPV does not make girls more promiscuous, study shows | <http://www.webcitation.org/6J2p9u3Eo> | 27 | 201 |
|  |  | 01-Oct-12 | HPV vaccine found to be safe in U.S. study of 190,000 women | <http://www.webcitation.org/6J2pCdmFt> | 14 |  |
|  |  | 18-Sep-12 | Toronto’s public health department expands HPV vaccine program for teen girls | <http://www.webcitation.org/6J2pFMTZt> | 1 |  |
|  |  | 25-Jan-12 | Ottawa recommends vaccinating males for HPV | <http://www.webcitation.org/6J2pK3X5s> | 1 |  |
|  |  | 03-Feb-12 | HPV vaccinations for boys urged by U.S. health authorities | http://www.webcitation.org/6J2pkVjsu | 2 |  |
|  |  | 06-Jan-12 | Lawrence Solomon: Get dirty and avoid vaccines | <http://www.webcitation.org/6J2pnWeqy> | 20 |  |
|  |  | 26-Jan-12 | Mouth, throat HPV infections more common among men: study | <http://www.webcitation.org/6J2prLBkF> | 5 |  |
|  |  | 28-Jun-12 | Opinion: Bishop Fred Henry defends Church stand on HPV vaccine | <http://www.webcitation.org/6J2q3Ru71> | 101 |  |
|  |  | 26-Jun-12 | Calgary bishop’s HPV vaccine ban putting thousands of girls at risk: MDs | <http://www.webcitation.org/6J2q6oOCW> | 425 |  |
